# Supplementary material for: Abstract rule learning promotes cognitive flexibility in complex environments across species
Source: Nat Commun. 2025 Jun 25;16:5396. doi: 10.1038/s41467-025-60943-7 (PMC12198366; doi:10.1038/s41467-025-60943-7)
Supplement: Supplementary file 1 — Supplementary Information [file 41467_2025_60943_MOESM1_ESM.pdf]

Supplementary Information

Abstract rule learning promotes cognitive flexibility in complex environments across species

Florian Bähner, Tzvetan Popov, Nico Boehme, Selina Hermann, Tom Merten, Hélène Zingone, Georgia Koppe, Andreas Meyer-Lindenberg , Hazem Toutounji, and Daniel Durstewitz

This PDF file includes:

Supplementary Methods

Supplementary Note 1 and 2

Supplementary Figure 1 to 10

Supplementary Table 1 to 9

Supplementary References

Supplementary Methods

Rat conventional set-shifting paradigm

In a paradigm adapted from Floresco et al.[1], the trial structure was as in the multidimensional rule-learning task. Rats first acquired a go-light rule (i.e., *press lever with illuminated cue light above*) until a performance criterion was reached (26/30 correct). Subsequently they switched back-and-forth between go-light and a place rule (*always press the lever on one side*). All rule switches were un-cued. A rat was considered to follow a given rule (i.e., having formed an attentional set) if it scored 18 correct trials out of 20. We used two versions of the task that differed with respect to reward feedback: reward feedback was either deterministic as in the original task version (two rule switches per session; min 30, max 200 trials per rule) or probabilistic (80/20% reward for correct/incorrect responses; one rule switch per session; min 30, max 250 trials per rule). The probabilistic version was introduced to encourage explorative choices and only used in implanted animals.

Rat empirical learning criterion

A classic performance criterion (such as 18 correct out of 20 responses) is not always a clear indication that a rat really followed a strategy consistent with the experimenter-defined rule. Increased reward rates for an incorrect strategy are possible in short blocks of trials because there are eight possible strategies but only two choices. We therefore defined an empirical learning criterion based on the length of a specific strategy sequence. We made use of the fact that strategies were also detected in an experimental cohort with random reward feedback. Strategy-specific

outliers with respect to sequence length were defined as values larger than the third quartile plus 1.5 times the interquartile range ($Q_3 + 1.5 \times (Q_3 - Q_1)$; based on 152 sessions with random reward feedback from 19 rats). We assumed that learning occurred if a detected strategy sequence length exceeded that threshold (i.e., it was an outlier). For example, for the *go-dark* strategy this threshold corresponds to a sequence length of 30 trials (Supplementary Table 4). Within the few sessions an animal needs to learn the go-dark rule, the onset trial of the first *go-dark* sequence exceeding this threshold in length is defined as the "learning trial" where the empirical learning criterion is reached.

Human learning criterion

Since the task was designed to both avoid ceiling effects and to fit into one MEG session (i.e., less trials per rule), we had to adapt our criteria for learning. In our MEG sample, humans tested the correct strategy at least once in 68.5% of cases. In order to decide whether human subjects actually learned a rule, two criteria were used. Learning criterion was either reached if the percentage of trials explained by the correct strategy exceeded 50% of all trials per rule or if PARCS (see next paragraph) detected a performance change point. This allowed us to also detect learning in cases where it occurred almost instantaneously (i.e., no performance change point could be detected) or rather late with only few trials left before the next rule switch (i.e., the percentage of trials in a rule explained by the correct strategy was low).

Change point detection

Formally, given a binary time series r_t describing whether the animal was rewarded ($r_t = 1$) or not rewarded ($r_t = 0$) in trial t, the CUSUM performance plot is given by $\mathrm{CUSUM}(r_t) = \sum_{t' \leq t}(r_{t'} - \langle r \rangle)$ where $\langle r \rangle$ is the mean of the entire time series. Candidate change points are located at extrema of the resulting curve. We used two different methods for statistical change point detection. For very short behavioral time series (rat two-rule deterministic set-shifting task), we used a sigmoidal learning-curve model, x_t, based on an inhomogeneous Bernoulli process[2] to detect changes in performance (with $y_t = 0$ and $y_t = 1$ corresponding to incorrect and correct responses at trial t, respectively),

$$\Pr(y_t = 1|x_t) = x_t^{y_t}(1 - x_t)^{1-y_t},$$

where,

$$x_t = b + \sum_i \frac{w_i}{1 + e^{-\frac{t-c_i}{\tau_i}}},$$

and c_i are the behavioral change points, i.e., the trials corresponding to a 50% change in correct response probability. Every sigmoid indexed by i corresponds to an individual learning ($w_i > 0$; increase in performance) or exploration ($w_i < 0$; decrease in performance) episode. The abruptness of the episode decreases with τ_i. These parameters are inferred using Maximum Likelihood Estimation. Sigmoidal learning curves were also used to visualize sudden transitions in performance (Figs. 1d, 2e-g, 5f-h).

For all other analyses, we used the Paired Adaptive Regressors for Cumulative Sum (PARCS) algorithm. The details of the procedure can be found in[3] and the MATLAB code is available at https://github.com/htoutounji/PARCS. The PARCS method requires a liberal guess of the number

of change points, followed by further refinement using non-parametric permutation bootstrap testing (significance level set to 0.05 based on 1000 bootstrap samples). PARCS has the advantage of detecting multiple change points in multivariate time series. However, compared to the Bernoulli approach, it is less sensitive to abrupt changes in binary, relatively short behavioral time series. In addition, we fitted a sigmoidal model to ± 100 trials centered on performance change points identified using PARCS to compute the 10-90% rise time as a measure for the abruptness of a performance change.

Reinforcement learning (RL) models

Task features, strategies and the s-a space: At every trial t we assume an animal makes a choice to press a lever based on current sensory cues in addition to place and reward history. There are thus four task features that can be probed for relevance: visual (relevant for the strategies *go-light* and *go-dark*), auditory (for *go-click* and *go-silent*), place (for *go-right*, *go-left*, and *alternate*), and outcome (for *win-stay-lose-shift*). The latter relates to place strategies as well, since the decision to stay or shift depends on both place and reward history. Further, it implicitly includes the opposite strategy of *win-shift-lose-stay*, which animals rarely followed.

Formally, a trial t can be fully described by a combination of current and last trial features called the state $\boldsymbol{s}_t$, a scalar response, or action $a_t \in \{L, R\}$, and scalar reward $r_t \in \{0,1\}$. The state is a four-dimensional binary vector $\boldsymbol{s} := [s_v, s_a, s_p, s_o]$, with each entry corresponding to one of the four task features. The sensory states $s_v, s_a \in \{L, R\}$ indicate whether the visual and auditory cues in the current trial are active on the left or right side of the operant chamber. The place state $s_p \in \{L, R\}$ indicates whether in the last trial the animal pressed the right or left lever. The outcome state

$s_o \in \{W, L\}$ indicates whether in the last trial the animal received a reward (win) or not (lose). The action $a \in \{L, R\}$ indicates which lever was pressed in the current trial. An outcome-dependent action is based on the place and outcome states, and indicates whether the animal pressed the same lever as in the last trial (stay), or switched to the other side (shift). As such, the eight strategies used to probe the four task features can be described by three s-a spaces as follows: The visual and auditory strategies are described by a 2×2 s-a space each. The place and outcome strategies are described by a 2-dimensional history-dependent state $\boldsymbol{s}_h \coloneqq [s_p, s_o]$, forming a 4×2 s-a space. More specifically, the mapping between strategies and state-action spaces is (Fig. 2a): *go-click*/*go-silent* (auditory cue), *go-light*/*go-dark* (visual cue), *alternate*/*go-left*/*go-right/win-stay-lose-shift* (place/outcome).

Attention-modulated RL models: In the rat cohort, we fitted two sets of attention-modulated RL models, one based on empirically obtained attention scores from video tracking data, and another based on binary attention scores. We first describe the first set of models and explain how they differ from second set below. We assume three relevant states, the third of which is a 2-dimensional state combining the place and outcome history features. For every state $i = 1, \dots, 3$ and trial t, the animal assigns values $Q_{ti}(s_{ti}, a_t)$ to each s-s pair that reflect the animal's expected reward from choosing action a_t at state s_{ti}, updates these values based on reward experience, and use them to guide future choices. To estimate expected reward in a trial, we assumed the animal combined values over states linearly, weighted by attention to the respective feature state at choice time,

$$Q_t(\boldsymbol{s}_t, a_t) = \sum_{i=1}^{3} w_{\mathrm{AC},ti} \cdot Q_{ti}(s_{ti}, a_t), \tag{1}$$

where $w_{\mathrm{AC},ti}$ are attention-at-choice scores obtained by normalizing raw angle values a_{AC} as measured by video tracking to the range [1/3, 1] with $\sum_{i=1}^{3} w_{\mathrm{AC},ti} = 1$ such that higher values correspond to stronger attention focus during choice.

In all models, values are updated through a Rescorla-Wagner-type learning rule[4],

$$Q_{t+1,i}(s_{ti}, a_{ti}) \leftarrow Q_{ti}(s_{ti}, a_{ti}) + \alpha w_{\mathrm{AL},ti} \delta_t, \quad (2)$$

where $\alpha \in [0,1]$ is a free parameter that modulates learning rate, $\delta_t := r_t - Q_t(\boldsymbol{s}_t, a_t)$ is a scalar reward prediction error, and $w_{\mathrm{AL},ti}$ are attention-at-learning scores that weigh the update for each task feature. Those scores are obtained by normalizing raw angle-sum values v_{AR} as measured by video tracking to the range [1/3, 1] such that higher values correspond to stronger attention focus during learning.

Given the linearly combined values over task features (Eq. 1), the policy, i.e., the probability of choosing an action given the current state, is computed using the softmax choice rule,

$$\pi(a_t|\boldsymbol{s}_t) = \frac{1}{1 + \exp\left(\beta\left(Q_t(\boldsymbol{s}_t, \bar{a}_t) - Q_t(\boldsymbol{s}_t, a_t)\right)\right)} \quad (3)$$

where $\beta \geq 0$ is a free parameter that modulates the balance between exploration and exploitation, and $\bar{a}_t$ is the opposite action of a_t (e.g., if $a_t = L$ then $\bar{a}_t = R$).

In the ACL model, learning and choice were modulated by empirically obtained attention measures $\boldsymbol{w}_{\mathrm{AL}}$ and $\boldsymbol{w}_{\mathrm{AC}}$, respectively. In the AC model, all $w_{\mathrm{AL},ti}$ were set to 1/3 such that value update was uniform across all rules. In the AL model, all $w_{\mathrm{AC},ti}$ were set to 1/3 such that choice in all rules was independent of attention. In the UA (uniform-attention) model, both $w_{\mathrm{AC},ti}$ and $w_{\mathrm{AL},ti}$ were set to 1/3 such that attention has no impact on neither learning nor choice. In all models, in trials where

attention was not clearly focused on a single strategy (no-attention trials), attention measures were set as in the UA model. Of note, since the attention measures were solely set according to the observed head direction angles, all models thus had the same number of free parameters (α, β).

In the human cohort, it was not possible to obtain empirical attention measures to different task features. However, we reasoned that our strategy-detection algorithm may indicate which task feature subjects are attending to in every trial and used binary attention measures in trials where a strategy is detected. For instance, in trials where the strategy *go-silent* or *go-click* was detected, both attention-at-choice and attention-at-learning scores for the auditory state space were set to 1 and those for the other two state spaces were set to 0. In trials where no strategy was detected, all scores were set to 1/3. To provide comparable findings across species, we report results of the binary attention models both in rats (Figs. 2d, 3f, g) and humans (Fig. 5e). In binary attention models, only part of the state space is relevant at any point in time, which corresponds to a strong filter that leads to dimensionality reduction. Empirical attention models (Supplementary Fig. 6a-c) allow a more graded filtering effect. However, both approaches led to the same results, which further validates the detection of low-dimensional strategies. We also used a variant of the binary ACL model to test whether all strategies are necessary in the model to predict held-out behavioral responses. More specifically, we compared the binary ACL model to three model variants where one of the s-a spaces (either auditory, visual or place-outcome) is treated as in the UA (uniform attention) model. This corresponds to a scenario in which strategy-specific attention effects for either auditory (*go-click*, *go-silent*), visual (*go-light*, *go-dark*) or place-outcome strategies (*alternate*, *go-left*, *go-right* and *win-stay-lose-shift*) are removed. Results for rats and humans performing multiple rule switches are shown in Supplementary Fig. 6d, e.

Standard RL models: We also consider three variations of standard RL models which directly map a multi-dimensional state to actions using 4-, 3-, or 2-dimensional state spaces and that are not modulated by attention scores. In the 4-dimensional state space model, the animal assigns value $Q_t(\boldsymbol{s}_t, a_t)$ to each combination of a state comprising all four relevant task features at the current trials and a binary action. This results in 32 different state-action values that are updated by a standard Rescorla-Wagner learning rule[4],

$$Q_{t+1}(\boldsymbol{s}_t, a_t) \leftarrow Q_t(\boldsymbol{s}_t, a_t) + \alpha\delta_t, \tag{4}$$

The probability of choosing an action given the current state, is computed using the softmax choice rule (Eq. 3). The 3-dimensional state space model excludes the outcome feature, resulting in 16 state-action values, while the 2-dimensional state space model excludes the place feature as well, depending only on sensory features and resulting in eight state-action values in total.

Model Inference, Comparison, and Simulation: In all four attention-modulated RL models, free model parameters α and β were inferred by maximizing data log-likelihood using MATLAB's constrained optimization, *fmincon*, implementing the active set algorithm,

$$\hat{\alpha}, \hat{\beta} \leftarrow \underset{\alpha\in[0,1],\beta\geq 0}{\operatorname{argmax}} \mathcal{L}_{\alpha,\beta}(\boldsymbol{s}, a, \boldsymbol{r}, \boldsymbol{w}_{AL}, \boldsymbol{w}_{AC}) = \underset{\alpha\in[0,1],\beta\geq 0}{\operatorname{argmax}} \sum_t \log \pi(a_t|\boldsymbol{s}_t). \tag{5}$$

Similarly for the three standard RL models, parameters were inferred by maximizing $\mathcal{L}_{\alpha,\beta}(\boldsymbol{s}, a, \boldsymbol{r})$. Model comparison used one-session forecasting for cross-validation. Each animal (N=29) attempted to maximize reward in M sequentially presented experimenter-defined rules (three or four rules with random rule sessions excluded). This required N sessions in total, where $N > M$ for all animals. For each experimenter-defined rule, all sessions up to and excluding the session where animal reached criterion were used as training data (free-parameter inference with maximum

likelihood). The last session per experimenter-defined rule is used for testing, where test-data likelihood is computed given the inferred parameters from the training data. This likelihood is then divided by the number of trials in the testing session to obtain trial-averaged likelihood. In total, M sessions were forecast per animal based on up to $N-1$ training sessions. The resulting M trial-averaged test likelihoods per model per animal were then averaged to compute a goodness-of-fit score and compared between the 7 RL models (Fig. 2d). A similar procedure was used to infer and compare models in human subjects (N=31; Fig. 5e), and the two cohorts of animals trained on a single experimenter defined rule (go-silent: N=19, alternate: N=19; Fig. 3b, c).

Based on the estimated model parameters, we simulated each of the seven model variants 1000 times. To generate the simulated model learning curves and their confidence intervals (Figs. 2e-g and 5f-h), we drew 100×100 samples of these 1000 simulations and computed the mean of each 100. This resulted in a 100 simulated mean-performance learning curves. The figures depict the median of these learning curves and the 90% confidence interval.

Rat electrophysiology: spike sorting

Preprocessed data were automatically spike sorted with Klusta (https://github.com/kwikteam/klusta) and afterwards manually curated with Klustaviewa (https://github.com/klusta-team/klustaviewa)[5]. Klusta uses a dual-threshold approach for automatic spike detection: events are detected as spatiotemporally connected components, in which the filtered signal exceeds a weak threshold for every point (2 times the standard deviation of the filtered signal) and at least one point is required to exceed a strong threshold (4 times the standard deviation of the filtered signal). This avoids both spurious detection of small noise events and

splitting of components of events into different clusters (i.e., if only some channels cross the strong threshold the signal on all other channels would be considered noise). During manual curation, each set of events (so-called “units”) detected by a particular template was first inspected and discarded if the events (“spikes”) comprising the unit were either judged to correspond to noise (i.e., non-physiological waveform shape or activity pattern across channels) or multi-unit activity (i.e., low-amplitude waveforms and/or multiple waveforms with refractory period contamination). The remaining units were compared to similar, spatially neighboring units to determine whether they should be merged (this was based on similarity of spike waveform/distribution across channels, drift patterns or cross-correlogram features). Similarly, a candidate unit was only kept if its spike waveforms were separated from all other units based on visual inspection of at least one 2D projection of principal component features. Finally, units were excluded if inter-spike interval violations exceeded 1% (i.e., more than 1% of consecutive spikes in accepted clusters with inter-spike interval less than 2ms). Units passing these criteria were considered to reflect the spiking activity of a neuron and included in further analyses.

Rat electrophysiology: single-unit analyses

In order to determine whether units are task-responsive, we tested whether firing rates/FR significantly discriminated between either different strategies, side of lever press, reward feedback or location of visual and auditory cues in each of the three trial phases (3s pre cue, 3s post cue, 3s post lever onset). Significance was assessed using Bonferroni-corrected unpaired t-tests with $p<0.05$. We also computed the selectivity index d’ for strategies during three trial phases for 3348 strategy pairs from 1884 units. d’ was computed as the absolute difference between the mean firing rates associated with the two sets of trials, divided by the square root of the sum of their variances.

This was done to assess how strong the strategy representations are at the single-unit level. d'>2 is required to reach a misclassification rate of less than 8% based on normality assumptions[6]. We computed the maximum FR change associated with a neural change point to exclude the possibility that population representations of strategies are related to strong FR changes of units across trials rather than their strategy selectivity (Supplementary Fig. 8f). More specifically, we used PARCS on the trial-wise spike count (for the entire trial from 3s pre cue to 3s post lever onset) of a unit to determine the location of neural FR change points. The maximum number of expected change points was set to three in the algorithm for all units. To assess how strong firing rates change during a change point, we used the following approach: for each CP_i we calculated the firing rate FR_{pre_i} from CP_{i-1} (or trial 1 for the first CP; $CP_{i=1}$) to CP_i, and the firing rate FR_{post_i} from CP_i to CP_{i+1} (or last trial for the last CP; $CP_{i=end}$). The strength of a change point was the absolute value of the FR changes from pre to post. In the case of several neural change points, the biggest FR change per unit was recorded and it was set to 0 if no FR change point was detected.

Rat electrophysiology: decoding analyses

Raw spike trains were first aligned to trial onset (spikes from -3s to +1s with respect to cue onset were extracted) for each prefrontal neuron. Trial-aligned spike trains from trials with a detected strategy were concatenated for each neuron and labelled accordingly (i.e., each trial had a label with the specific strategy used). Spikes were then binned using a sliding window approach. We systematically tested the effect of different bin widths [250, 500, 1000, 2000, 3000ms] and respective step sizes [25, 50, 100, 200, 300ms] on decoding accuracy. We used z-score normalized data to avoid that neurons with higher firing rates have a larger influence on the classification results. We used a maximum-correlation-coefficient[7] classifier for decoding analyses based on

leave-one-out cross-validation. The classifier learns a mean neural population vector for each class (i.e., a template for a detected strategy in our case) by averaging all training points within each class. The classifier predicts the correct class using Pearson's correlation coefficient between the test data and the training set of each class (the highest correlation value corresponds to the predicted label). In our case, neural data from 14 trials of each strategy were used as a training set to predict which strategy a rat used in the test trial. Decoding accuracy for each session was based on 50 cross-validation runs using a resampling procedure. Accuracy on average increased monotonically [71.7 (66.4-79.6), 74.3 (66.9-83.1), 76.3 (68.9-84.7), 79.5 (69.8-88), 80 (69.1-88.3) %] and was maximal for the coarsest bin width/step size in 45/62 cases. All follow-up analyses were thus performed using the bin width with the best decoding results across sessions (3000ms bin width/300ms step size) and the decoding analysis was repeated with a bigger time window (-20s to +6s centered on cue onset to cover all task stages and the entire inter-trial interval) to determine the time point of maximum decoding accuracy.

In order to make decoding accuracies comparable between sessions with different number of detected strategies (range: 2-4 per session), we performed all further decoding analyses using strategy pairs (N=105) with the same analysis parameters. We performed two further analyses to show that high SNR for strategy pairs at the population level depends on small contributions from multiple, strategy-selective units. First, we estimated the contribution of individual units using a so-called neuron dropping procedure[7,8]. More specifically, we repeated strategy decoding at the population level but systematically dropped one unit at a time and then compared the decoding accuracy based on all units in a session with the accuracy after removing one unit. Negative values indicate a decrease in decoding accuracy (Supplementary Fig. 8e). We also ranked all units in a

session according to d' strategy in each trial phase and repeated population decoding after removing the top 5, 10, 20, 30 or 40% units from the strategy decoding analysis (Supplementary Fig. 8g).

In some analyses we compared how decoding of strategies compares to decoding of other task variables (Fig. 4e) or state-action pairs as defined in RL models (Fig. 4f) within a session. For these analyses, we used a normalized decoding score because the decoded categories differed with respect to the number of classes per behavioral category (i.e., decoding accuracy was normalized such that a score of 0 corresponds to chance level and a score of 100 to perfect decoding). Given that there are up to 32 different state-action-pairs (2D-RL: 8, 3D-RL: 16 and 4D-RL: 32 state-action-pairs) and only 300 trials per session, not all classes could be decoded from each session. We decided to use only sessions that had at least 15 trials for at least four different state-action-pairs (N=39 sessions).

We also conducted so-called generalization analyses which test whether decoded neural representations are abstract[7]. This approach was used for two analyses.

First, we used a subset of sessions (using pairs with at least 30 trials per strategy) to test whether the prefrontal representation of strategies (that can be interpreted as abstract actions[9]) depends on the motor response in a given trial (i.e., left versus right lever press) or is invariant with respect to that feature (Fig. 4g). More specifically, we trained the classifier on the neural data (either 3s before cue onset, after cue onset or following lever onset) of 14 trials of each strategy with a motor response on one side and compared the decoding accuracy in the test set when using trials with a motor response on either the same or the opposite side (50 resample runs).

Second, we decoded whether rats pressed the right or the left lever and tested whether this representation depended on a specific task feature (Supplementary Fig. 9). For example, to test whether action decoding depends on the task feature auditory cue, all right and left choice trials of

a session were sorted according to whether the right or left loudspeaker was active in a given trial. We then trained the decoder on left-right choice pairs (neural data either from 3s before cue onset, after cue onset or following lever onset) for which the loudspeaker was active on the same side and tested on pairs with the active loudspeaker on the same or the opposite side. We used the same analysis parameters as above (at least 15 trials for each of the four conditions, 50 resample runs). Action decoding was defined as task feature-dependent if decoding accuracy was higher in the condition where the side of the active loudspeaker is the same in both the train and test condition. All trials of a session were used with the exception of sessions in which the empirical learning criterion was reached. In this case, all trials that occurred before the learning criterion were included in the analysis.

Supplementary Note 1. Evidence for *de novo* strategy formation in rats.

Some strategies (e.g., anti-cue strategies) were only detected if they corresponded to the current task rule but rarely spontaneously, even if rats learned multiple rules (see Fig. 3g, Supplementary Fig. 7a). This indicates that new strategies are learned if the behavioral repertoire does not include the strategy that corresponds to the current task rule. We reasoned that strategy formation requires that rats sample the relevant state-action space first to learn correct mappings at the low level. This will increasingly affect action selection and eventually a novel high-level strategy is formed. To test this assumption, we first tried to identify behavioral markers related to action selection that predict the learning trial. Indeed, the performance change point does not only correlate with learning (Fig. 1e) but also precedes the empirical learning criterion both in rats learning one rule and multiple rules (Supplementary Fig. 7b). We also used change point analysis to test if attention increases during rule acquisition and whether such transitions are predictive of learning. While attention-at-choice in RL models reflects trial-by-trial fluctuations when rats focus their attention on different task features, this analysis focused on how attention for the strategy-specific task feature changes during rule learning. Indeed, we detected transitions in attention measures in a large proportion of learned rules (attention-at-choice with respect to the strategy-specific feature: 88.1%, attention-at-reward: 66.1%). In line with the idea that strategy formation requires increased learning, the first correct strategy sequence occurred much later in rules for which an attention-at-reward change point could be detected (trial 414 (182.3-817.8), N=72 rules) as compared to rules without such a change point (trial 53 (34-257), N=37 rules; $p=9*10^{-6}$, two-sided Mann-Whitney test). We then estimated a linear regression model to show that both attentional change points (attention-at-choice $p=1.9*10^{-15}$, attention-at-reward $p=7.3*10^{-4}$) predict the learning trial with high accuracy ($R^2_{adjusted}=91.5\%$; Supplementary Fig. 7c). Change points for attention-at-reward

preceded both attention-at-choice change points and the learning trial but the latter two were not different (Supplementary Fig. 7d).

Supplementary Note 2. Abstract representation of task features in rat PFC.

We used a similar analysis approach as in Fig. 4h to test whether there was electrophysiological evidence that rats have a representation of the cue that is relevant for the current task rule even before they infer the correct rule (i.e., while they are sequentially testing different hypotheses). We decoded whether rats pressed the right or the left lever and evaluated whether decoding accuracy depended on the task feature that is relevant for the current task rule (Supplementary Methods). More specifically, in the example given in Supplementary Fig. 9a, all right and left choice trials of a session (i.e., no matter whether trials were assigned to a specific strategy or not) were sorted according to whether the right or left loudspeaker was active in a given trial. We then trained the decoder on left-right choice pairs for which the loudspeaker was active on the same side and tested on pairs with the active loudspeaker on either the same or the opposite side. We reasoned that action decoding is dependent on a task feature if decoding accuracy is significantly higher in the condition where the side of the active loudspeaker is the same in both the train and test condition. We found that action decoding depended on the relevant task feature (e.g., sound for rats learning the rule go-silent) in the three seconds following cue and lever onset (Supplementary Fig. 9b).

Furthermore, this decoding asymmetry (i.e., the difference in decoding accuracy between same vs. different) was specific for the task-relevant feature. We showed this in two different ways. First, we compared the difference between the decoding accuracy for the relevant task feature compared to the median of all other features. We did not detect a significant difference for the three seconds

prior to cue onset. However, we found higher decoding asymmetry for the relevant task feature during cue presentation and after lever onset (Supplementary Fig. 9c). Second, we counted how often the decoding difference was >5% for the relevant task feature vs. all other features. Again, there was no difference for the three seconds prior to cue onset (7/41 vs. 16/115 sessions with >5% difference, $p=0.62$, chi-square test) but for the three seconds post cue (19/41 vs. 27/115, $p=0.0058$) and post lever onset (38/41 vs. 22/115, $p=1.1*10^{-16}$).

Supplementary Fig. 1. Rats also use behavioral strategies in an established two-rule set-shifting task. Related to Fig. 1.

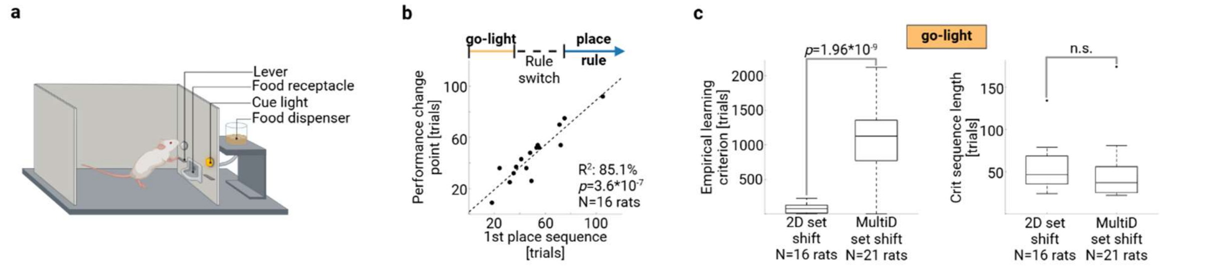

a Rats learned to switch from a go-light rule (i.e., *press lever with illuminated cue light above*) to a place rule (*always press the lever on one side*) in a classic operant set-shifting task[1]. We also used our strategy detection algorithm and assumed that rats may use up to six different behavioral strategies in this task (*go-light/go-dark*, *go-right/go-left*, *alternate or win-stay-lose-shift*). Indeed, multiple behavioral strategies (including the strategy corresponding to the experimenter-defined rule) were detected during initial rule learning (3 (2-3) strategies tested during initial go-light rule, N=16 rats). **b** In this task, sudden transitions in performance are known to occur during a first rule switch from a go-light to a place rule[6]. Our results indicate that these transitions can be explained by strategy switches: the scatter plot shows a significant correlation between the performance change point and the onset of the first correct *place* strategy sequence (Pearson correlation). These results are thus compatible with our hypothesis that rats follow low-dimensional behavioral strategies to infer task rules. However, it remains unclear whether the detected strategies are relevant for learning because the learning curve is rather short (performance change point occurred already after 46.3 ± 5.2 trials) and the state space of this task is very simple (two states are sufficient). **c** We compared how fast naive rats acquire the rule go-light either in the multidimensional rule learning or the conventional operant rule learning task. Indeed, rats in the

multidimensional task were significantly slower to reach the learning criterion (sequence length length ≥22 trials, two-sided unpaired t-test; Supplementary Table 4) but the length of *go-light* sequences when reaching criterion were not different (p=0.33, two-sided Mann-Whitney test) which indicates a similar performance in both groups. Box plots showing median, 25%–75% percentile, whiskers: 1.5 IQR and outliers. Created in BioRender. Böhme, N. (2025) https://BioRender.com/j03r892. Source data are provided as a Source Data file.

Supplementary Fig. 2. Head direction plots during choice formation for all strategies detected in the multidimensional rule-learning task. Related to Fig. 2.

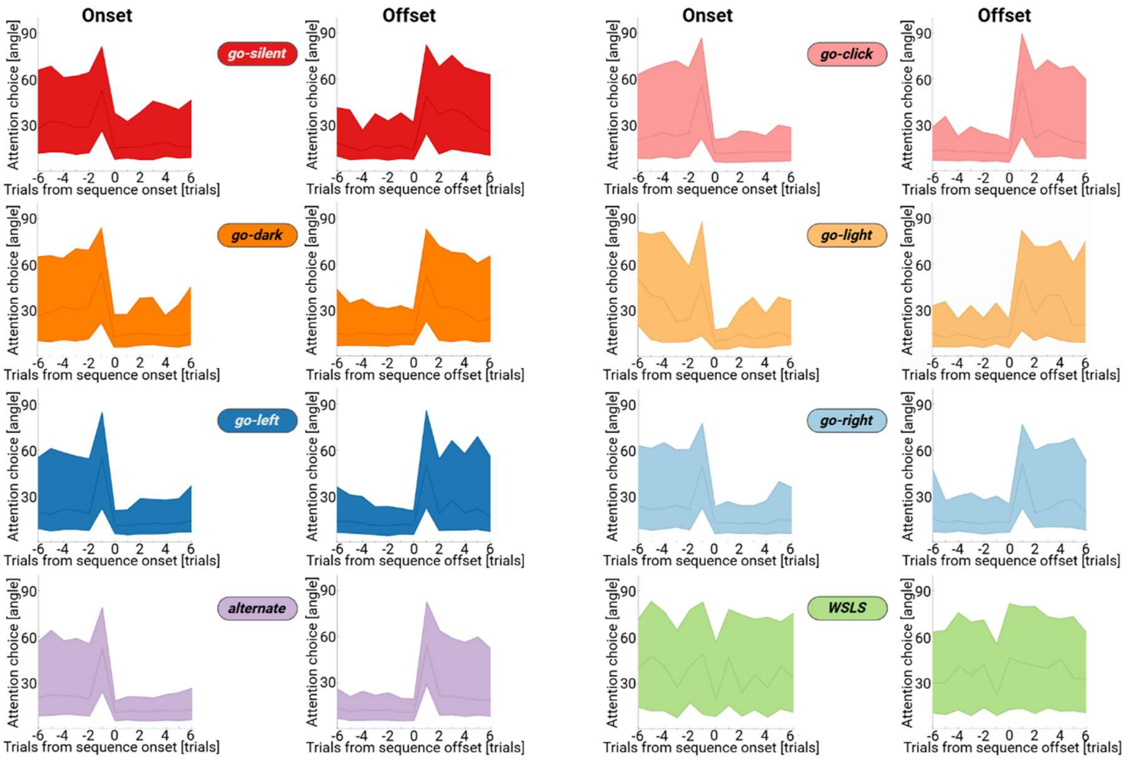

Sudden transitions in choice at sequence onset/offset were related to attention shifts for all eight detected strategies. Head-direction plots show head angles at cue onset with respect to the correct side (e.g., the side with the active cue light for the *go-light* strategy) are shown for the six trials before/after strategy onset/offset. Note that the strategy-specific head angles abruptly decreased/increased at strategy onset/offset (corresponding to trial 0). Each plot is the average of multiple strategy sequences (as detected using our strategy detection algorithm) from 29 rats performing several rule switches in the multidimensional rule-learning task. The table below lists how many sequences were detected per behavioral strategy and the p-value (two-sided Wilcoxon matched pairs test; Supplementary Table 1) for the decrease in head angle at strategy onset

(compared to the trial before, i.e., trial -1 vs. trial 0) and the increase at strategy offset (last trial of sequence vs. trial after, i.e., trial 0 vs. trial +1). Data are presented as median, first and third quartile. Created in BioRender. Böhme, N. (2025) https://BioRender.com/n06m549. Source data are provided as a Source Data file.

Supplementary Table 1. Related to Supplementary Fig. 2.

Strategy	Number of sequences	Decrease in head angle at strategy onset	Increase in head angle at strategy offset
alternate	578	$1.1*10^{-66}$	$6.6*10^{-64}$
go-click	648	$4.2*10^{-52}$	$5.9*10^{-59}$
go-dark	593	$8.6*10^{-42}$	$6.9*10^{-45}$
go-left	519	$1.2*10^{-53}$	$6*10^{-54}$
go-light	99	$2.9*10^{-10}$	$2.4*10^{-8}$
go-right	330	$1.1*10^{-29}$	$4.1*10^{-25}$
go-silent	210	$3*10^{-15}$	$2.6*10^{-17}$
WSLS	139	$2.5*10^{-3}$	0.79

Supplementary Fig. 3. Head direction plots during choice formation for all strategies detected in naïve rats receiving random reward feedback. Related to Figs. 2, 3.

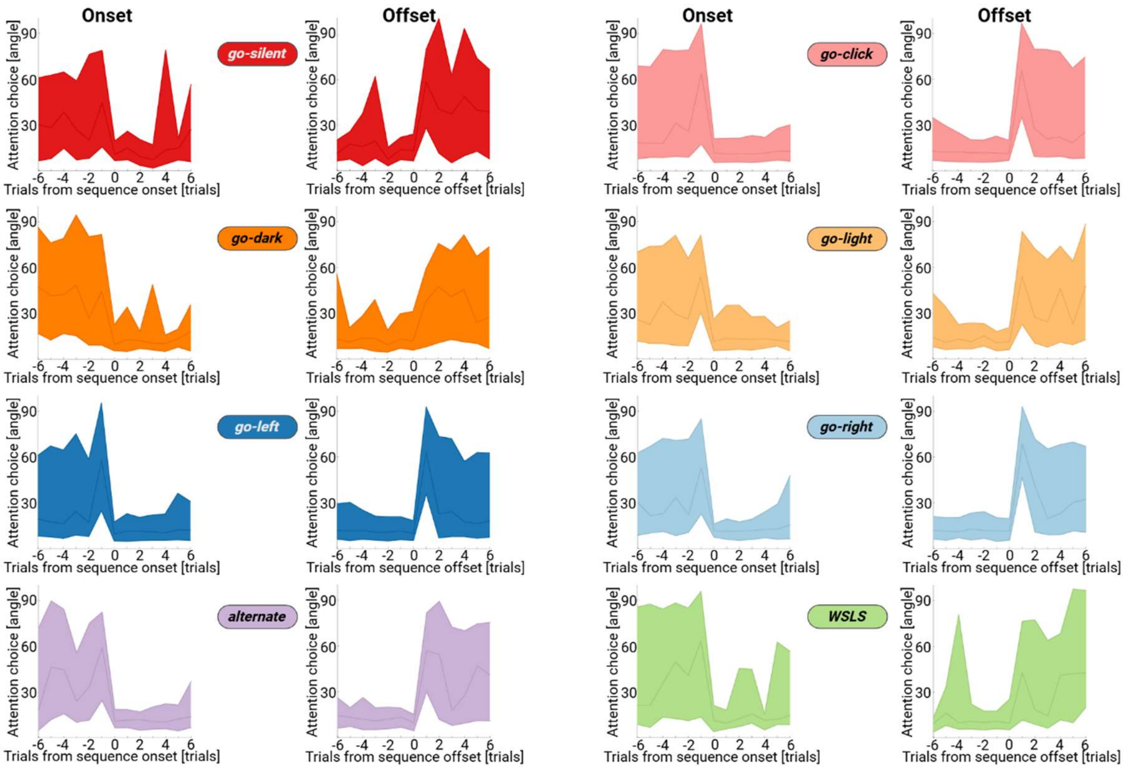

Similar plots as in Supplementary Fig. 2 but this time the head direction plots are based on the average of multiple strategy sequences from 19 experimentally naïve rats performing eight sessions each with randomized reward feedback (i.e., without a task rule). This is interesting given that task feature-based learning in the absence of a clear behavioral advantage has also been reported in the context of human multidimensional learning[10,11]. Statistics for head angle decrease/increase at strategy onset/offset are given below (two-sided Wilcoxon matched pairs test; Supplementary Table 2). Data are presented as median, first and third quartile. Created in BioRender. Böhme, N. (2025) https://BioRender.com/z08u535. Source data are provided as a Source Data file.

Supplementary Table 2. Related to Supplementary Fig. 3.

Strategy	Number of sequences	Decrease in head angle at strategy onset	Increase in head angle at strategy offset
alternate	75	$3.7*10^{-9}$	$5.9*10^{-9}$
go-click	432	$1.7*10^{-40}$	$3.6*10^{-47}$
go-dark	38	$9.6*10^{-3}$	0.03
go-left	353	$1.6*10^{-37}$	$8.3*10^{-42}$
go-light	102	$8.9*10^{-11}$	$2.8*10^{-11}$
go-right	135	$1.1*10^{-16}$	$4.5*10^{-17}$
go-silent	18	$5*10^{-3}$	$8.6*10^{-4}$
WSLS	38	$9.9*10^{-5}$	$9.9*10^{-5}$

Supplementary Fig. 4. Head direction plots during choice formation for all strategies detected in experienced rats receiving random reward feedback. Related to Figs. 2, 3.

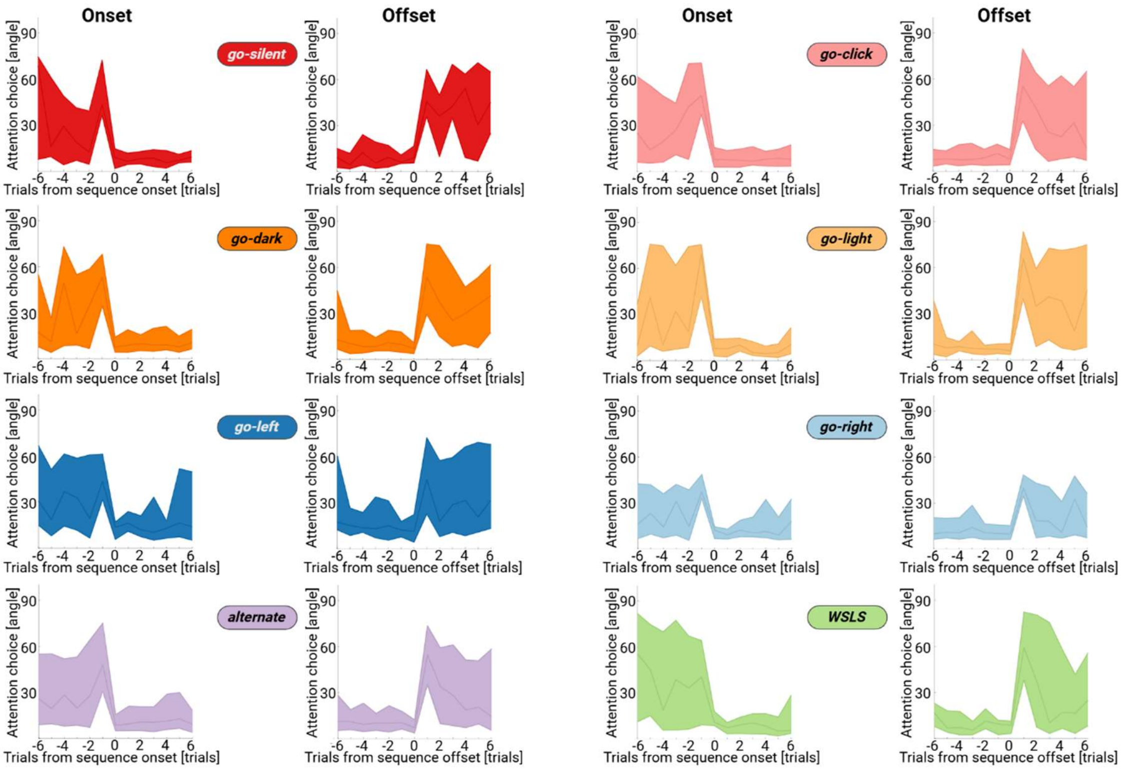

Similar plots as in Supplementary Figs. 2, 3. Head direction plots are based on the average of multiple strategy sequences from eight experienced rats performing five sessions each with randomized reward feedback (i.e., without a task rule). Those rats learned a sequence of rules (go-dark→ place→ alternate) before the random rule sessions started. Statistics for head angle decrease/increase at strategy onset/offset are given below (two-sided Wilcoxon matched pairs test; Supplementary Table 3). Data are presented as median, first and third quartile. Created in BioRender. Böhme, N. (2025) https://BioRender.com/n58x957. Source data are provided as a Source Data file.

Supplementary Table 3. Related to Supplementary Fig. 4.

Strategy	Number of sequences	Decrease in head angle at strategy onset	Increase in head angle at strategy offset
alternate	89	$8*10^{-13}$	$9*10^{-12}$
go-click	52	$1.1*10^{-5}$	$5.3*10^{-9}$
go-dark	37	$6.2*10^{-6}$	$2.1*10^{-6}$
go-left	35	$4.2*10^{-6}$	$3.1*10^{-4}$
go-light	21	$4.2*10^{-4}$	$6.9*10^{-5}$
go-right	54	$1.9*10^{-7}$	$1.1*10^{-8}$
go-silent	13	$7.3*10^{-4}$	0.022
WSLS	19	0.014	$2.9*10^{-4}$

Supplementary Fig. 5. Movement plots during the inter-trial interval (ITI) for strategies detected in rats learning multiple rules or receiving random reward feedback. Related to Figs. 2, 3.

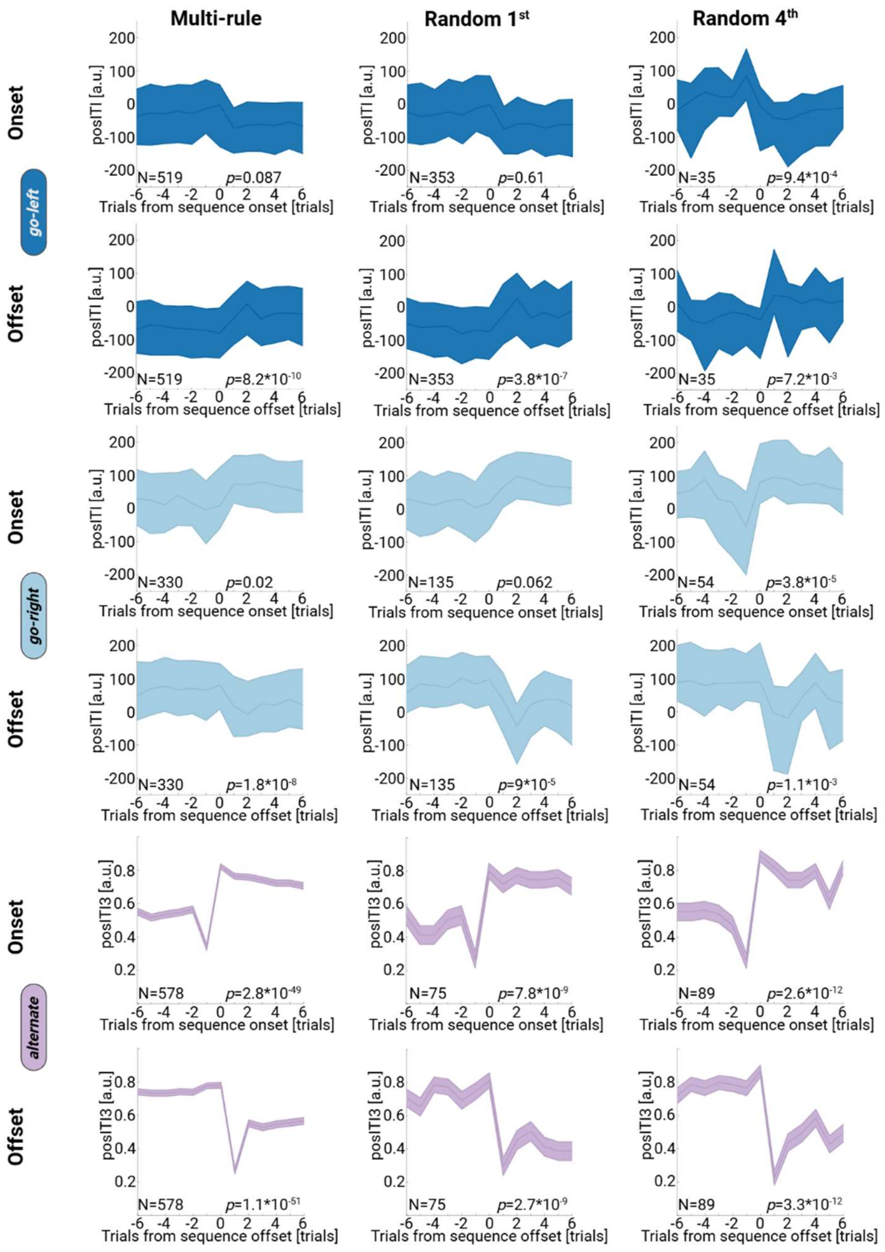

For three strategies (*go-right*, *go-left* and *alternate*), we also found strategy-specific movement patterns even before cue onset which indicates that rats focus their attention on a single task feature

(Methods). More specifically, we found that rats tend to stay in the respective side of the operant chamber at the onset of a *place* strategy and move away directly after a sequence stops (posITI). Similarly, rats switch sides in the inter-trial interval while they follow the *alternate* strategy (posITI3). We detected this effect in three different data sets (Supplementary Figs. 2-4). This indicates a strong top-down process such that the attention selection process is already completed before cue onset. Plots are similar to head-direction plots, but instead of the head angle we show movement parameters in the ITI for the six trials before/after strategy onset/offset. Each panel lists how many sequences were detected per behavioral strategy and the p-value (two-sided Wilcoxon matched pairs test) for the movement parameter change at strategy onset (compared to the trial before, i.e., trial -1 vs. trial 0) and at strategy offset (last trial of sequence vs. trial after, i.e., trial 0 vs. trial +1). Data are presented as median, first and third quartile (*go-right, go-left*) or mean ± sem (*alternate*). Created in BioRender. Böhme, N. (2025) https://BioRender.com/g01c909. Source data are provided as a Source Data file.

Supplementary Fig. 6. Model comparisons with variants of attention-modulated RL models. Related to Figs. 2, 3, 5.

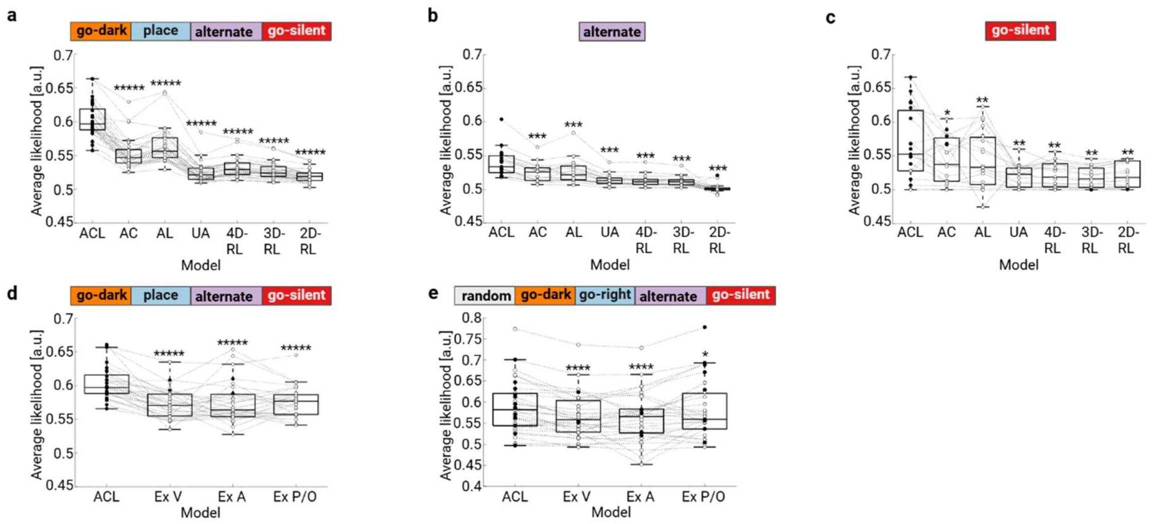

a Trial-averaged cross-validated likelihood for seven RL model variants with continuous attention scores (i.e., head movements) instead of binary scores which are based on data from 29 rats that learned a sequence of rules (related to Fig. 2d). **b, c** Replication of RL model findings using empirical attention scores for rats learning alternate (N=19; **b**) and go-silent (N=19; **c**) as a first rule (related to Fig. 3b, c). **d, e** Model comparisons between binary ACL model and three model variants where one of the s-a spaces (either auditory - Ex A, visual - Ex V or place-outcome - Ex P/O) is treated as in the UA (uniform attention) model. Comparisons are based on data from 29 rats (**d**) and 31 humans (**e**) that learned a sequence of rules (related to Figs. 2d, 5e). Removal of strategy-specific attention effects related to any of the s-a spaces resulted in worse prediction of held-out behavioral responses. Box plots in all panels show median, 25%–75% percentile, whiskers: 1.5 IQR and outliers; dashed lines connect values (circles) of the same individual; filled circles represent best (i.e., highest likelihood) model of each individual. Model comparisons between ACL and all other models are Benjamini-Hochberg corrected (two-sided Wilcoxon matched pairs test

with *: $p<0.05$, **: $p<0.01$, ***: $p<10^{-3}$, ****: $p<10^{-4}$, *****: $p<10^{-5}$). Created in BioRender. Böhme, N. (2025) https://BioRender.com/k27n333. Source data are provided as a Source Data file.

Supplementary Fig. 7. Formation and value-based selection of behavioral strategies in rats. Related to Fig. 3.

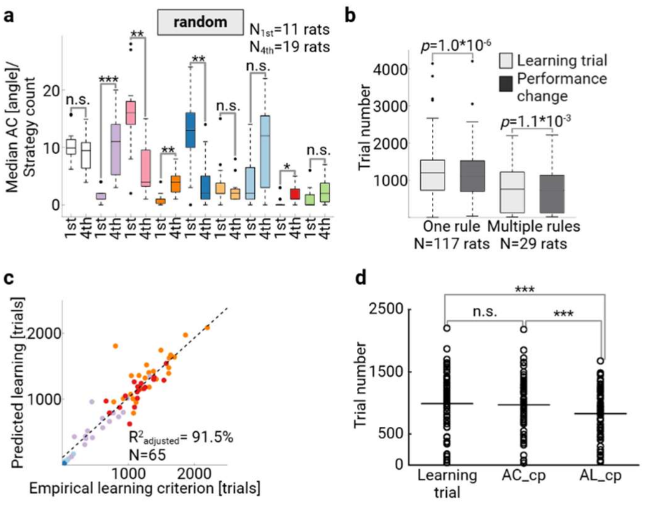

a Comparison of naïve (N=19) and experienced (N=11) rats performing the random rule (1505 trials per rat). Median attention-at-choice values were not different between groups (p=0.37, two-sided Mann-Whitney test). However, groups differ with respect to how often they select specific strategies (strategies are color-coded as in Fig.1): *alternate*: $p=1.1*10^{-4}$, *go-click*: $p=3*10^{-3}$, *go-dark*: $p=3*10^{-3}$, *go-left*: $p=9*10^{-3}$, *go-light*: p=1, *go-right*: p=0.26, *go-silent*: p=0.018, *win stay-lose shift*: p=0.23 (two-sided Mann-Whitney tests, Bonferroni-corrected for multiple comparisons). This analysis indicates that strategy selection is modulated by learning experience. Moreover, within the group of naïve rats strategy selection was not equiprobable (Friedman test, $p<10^{-4}$) with a preference for cue and place over anti-cue strategies. Box plots showing median, 25%–75% percentile, whiskers: 1.5 IQR and outliers. **b** Performance change points precede the learning trial both in rats learning one (N=117) or multiple rules (N=29), two-sided Wilcoxon signed-rank test. Box plots showing median, 25%–75% percentile, whiskers: 1.5 IQR and outliers. **c** Abrupt changes in attention (during choice formation and reward feedback) across trials predict learning ($R^2_{adjusted}$).

Change points (N=65) were detected using PARCS (Methods). Each dot represents the empirical vs. predicted learning trial. Rules are color-coded as in Fig. 1. **d** Dot plots (horizontal line indicates group mean) show that attention-at-reward change points (AL_cp) preceded both attention-at-choice change points (AC_cp) and the learning trial (repeated-measures ANOVA, $p<10^{-4}$, F=18.7, Bonferroni's multiple comparison test with ***: $p<10^{-3}$ for significant pairs). Created in BioRender. Böhme, N. (2025) https://BioRender.com/w58r889. Source data are provided as a Source Data file.

Supplementary Fig. 8. Single-unit representations in rat PFC. Related to Fig. 4.

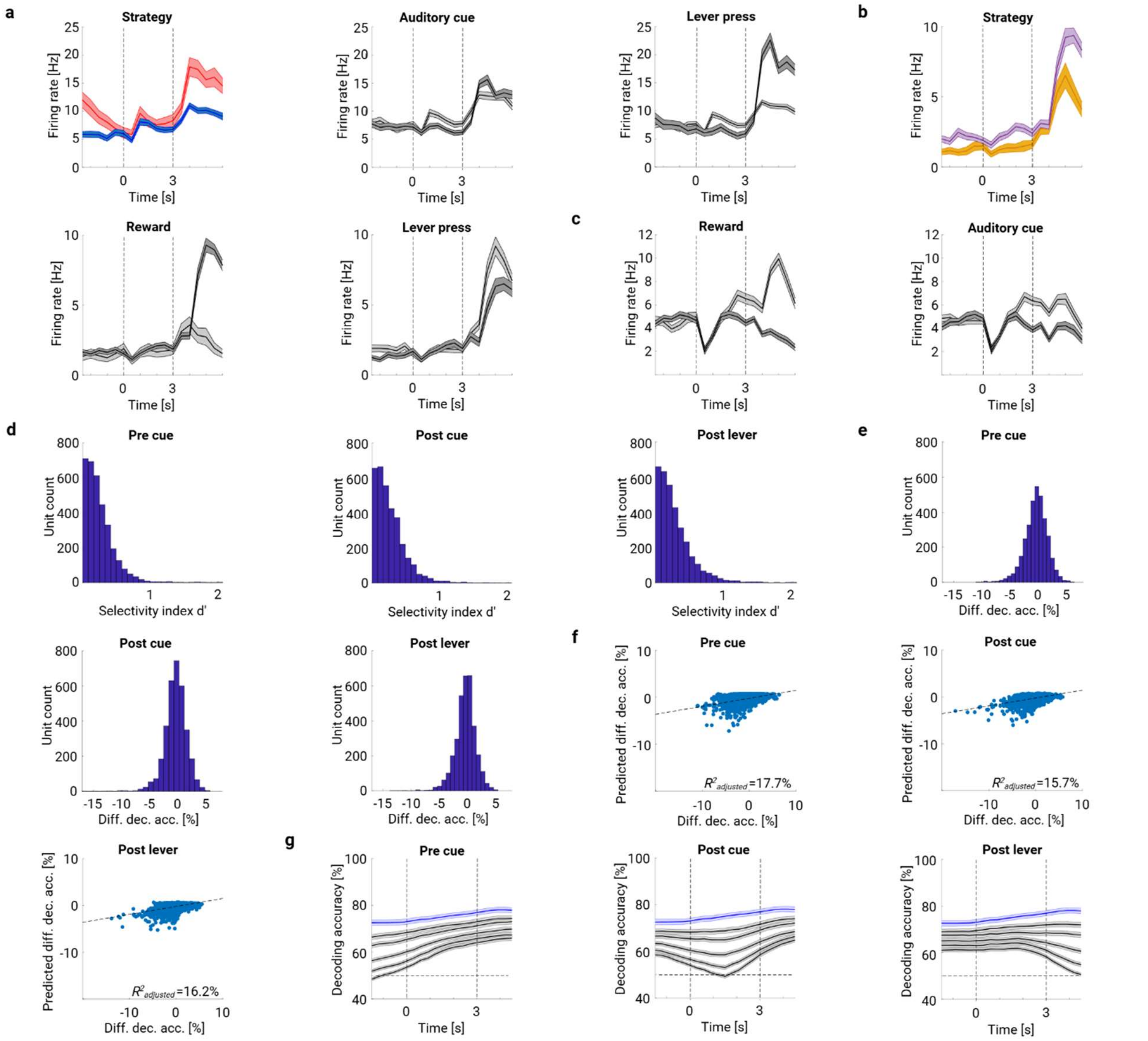

a-c PSTHs of example units with mixed selectivity (mean values +/- SEM; Bonferroni-corrected, two-sided, unpaired t-tests). **a** Unit with 9.2 Hz mean firing rate/FR that significantly discriminated between strategies (*go-click*/red, *go-left*/blue; $p_{pre\ cue}=8.5*10^{-3}$, $p_{post\ lever}=5.2*10^{-8}$), auditory cue location (left side/light grey, right/dark grey; $p_{post\ cue}=0.046$) and side of lever press (left/light grey, right/dark grey; $p_{post\ cue}=0.04$, $p_{post\ lever}=2.5*10^{-19}$). **b** Unit with 2.3 Hz mean FR that responded to strategies (*alternate*/purple, *go-dark*/orange; $p_{pre\ cue}=0.024$, $p_{post\ cue}=9.1*10^{-3}$, $p_{post\ lever}=7.3*10^{-4}$),

reward feedback (no reward/light grey, reward/dark grey; $p_{post\ lever}=7*10^{-11}$) and side of lever press (left/light grey, right/dark grey; $p_{post\ lever}=0.02$). **c** Unit with 4.3 Hz mean FR that significantly discriminated between reward feedback (no reward/light grey, reward/dark grey; $p_{post\ lever}=10^{-33}$), auditory cue (left side/light grey, right/dark grey; $p_{post\ cue}=2*10^{-3}$, $p_{post\ lever}=8.2*10^{-7}$). **d** Distribution of d' strategy during three trial phases based on 3348 strategy pairs from 1884 units indicates a low SNR. d'>2 is required to reach a misclassification rate <8% based on normality assumptions[6] and was only observed in 3/1884 units. **e** Comparison of decoding based on all units in a session with the accuracy after sequentially removing one unit at a time. This difference in accuracy is shown for all trial phases. Negative values indicate decreased accuracy after unit removal. **f** Scatter plot of regression models: each dot represents the empirical vs. predicted accuracy difference. Differences were only predicted by d' ($p_{pre\ cue}=4.2*10^{-117}$, $p_{post\ cue}=2*10^{-106}$, $p_{post\ lever}=2.8*10^{-105}$). Neither strong FR changes across trials (measured as the unit's maximum FR change associated with a neural change point, all $p>0.05$; Supplementary Methods), total neuron number recorded in a session ($p_{pre\ cue}=0.74$, $p_{post\ cue}=3.4*10^{-3}$, $p_{post\ lever}=0.06$), nor number of task features coded by a unit ($p_{pre\ cue}=0.02$, $p_{post\ cue}=0.94$, $p_{post\ lever}=0.55$) were consistent predictors. **g** Units were ranked according to d' in each trial phase and population decoding was repeated after removing the top 5, 10, 20, 30 or 40% units (accuracy across time shown as mean ± sem for 105 strategy pairs, blue line corresponds to decoding based on all units). Only after removing the top 40% of units, accuracy was not higher than 50% chance level (one-sample t-test with $p>0.05$) and this effect was restricted to the respective trial phase. Created in BioRender. Böhme, N. (2025) https://BioRender.com/hnomsfl. Source data are provided as a Source Data file.

Supplementary Fig. 9. Abstract representation of task features in rat PFC. Related to Fig. 4.

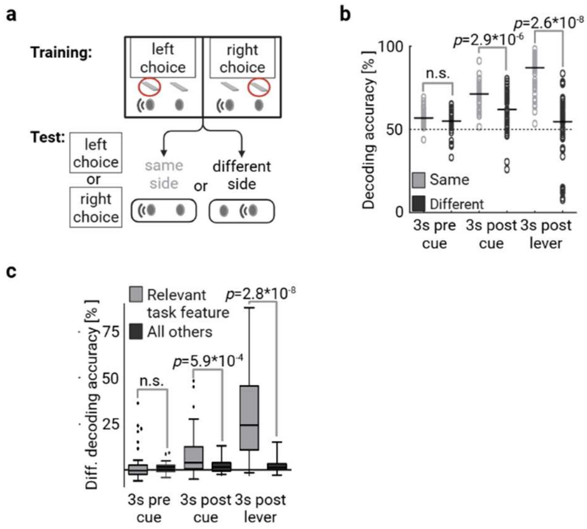

a Analysis strategy to test whether action decoding depends on a specific task feature (example sound). **b** Action decoding depended on the relevant task feature, most prominently following lever onset (N=41, dot plot with median as horizontal line, two-sided Wilcoxon matched pairs test). The dotted horizontal line at 50% corresponds to chance level. Decoding accuracy was above chance in all conditions (all $p<10^{-4}$, two-sided unpaired t-tests not corrected for multiple comparisons) with the exception of the last condition (i.e., data at the time of lever press with different task feature in training and test; p=0.95). **c** The difference in decoding accuracy between same and different was plotted for three consecutive time bins both for the rule-relevant task feature and the median of all other task features. Decoding asymmetry was task feature-specific for both the three seconds post cue and lever onset (N=41, two-sided Wilcoxon matched pairs test). Box plots showing median, 25%–75% percentile, whiskers: 1.5 IQR and outliers. Created in BioRender. Böhme, N. (2025) https://BioRender.com/q15c698. Source data are provided as a Source Data file.

Supplementary Fig. 10. MEG evidence for strategies and related attention focus. Related to Fig. 5.

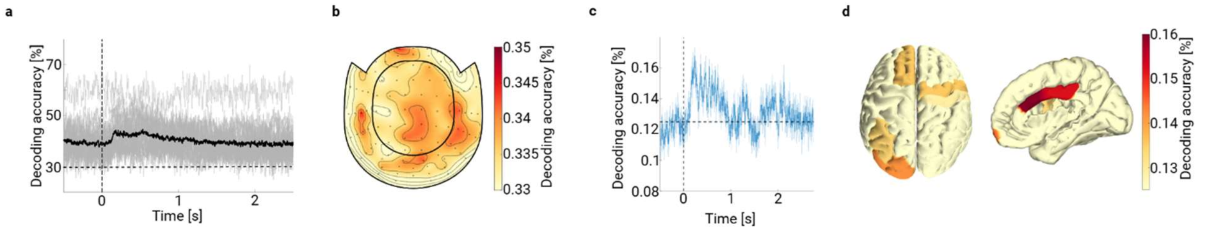

a Trial-based decoding accuracy of the current attention focus across time (related to Fig. 5i). Overlay of all 28 individual decoding curves, black curve corresponds to mean (vertical dashed line: cue onset, horizontal dashed line: 33% chance level). **b** Sensor-level topography of attention focus decoding (averaged over time window of 180 to 530 ms following stimulus onset in Fig. 5i). **c, d** Strategy decoding based on a source-level fixed effects analysis. Given that the data was expressed within the same geometrical position (i.e., parcels) within the same coordinate system, we pulled all trials of all subjects first. This resulted in a data matrix consisting of 11179 samples, 68 parcels, 976 time points, and 8 classes. This procedure resulted in the following distribution of class frequencies: 12.99% [go-light], 17.48% [go-dark], 17.45% [go-click], 21.50% [go-silent], 12.00% [go-right], 1.57% [go-left], 14.41% [alternate], 2.61% [win-stay lose-shift]. Decoding across time is shown in **c** and the source-level topography of the highlighted decoding peak (averaged over selected time range: 180-280 ms after cue onset) in **d**. The decoding peak was located in the right cingulate (caudal anterior division and posterior cingulate). In the context of rule learning and switching, anterior cingulate seems to be involved in monitoring the reliability of the current behavioral strategy[12]. Significant decoding was also detected in left superior frontal gyrus, right precentral gyrus, right postcentral gyrus, left inferior parietal cortex and left lateral occipital cortex. Created in BioRender. Böhme, N. (2025) https://BioRender.com/e31s308.

Supplementary Table 4. Strategy parameters for rats learning one rule in the multidimensional rule-learning task. Related to Fig. 1.

Rule (N)	learn tresh [n]	sequences [n]****	y [t]****	perf cp [t]****	seq long [n]****	x_1 [t]****	x_2 [seq]**	x_3 [t]****	om [t]**
Alternate (19)	30	50.9 ± 4	1090 ± 116.8	957.3 ± 96.2	69 (52-97)	459.5 ± 65.5	4.1 ± 0.6	1150 ± 95	126 (25-256)
Go-click (17)	58	20.8 ± 4.1	462 ± 97.6	346.7 ± 77.11	116 (96-160)	26.3 ± 14.4	6.5 ± 1.5	n.a.	6 (3-43.5)
Go-dark (24)	30	46.3 ± 3.3	1336 ± 77.8	1308 ± 66.3	191 (141.3-223.8)	709.3 ± 86	4 ± 0.2	1091 ± 58.6	18 (7-82.75)
Go-light (21)	22	47.8 ± 2.8	1064 ± 105.4	1115 ± 79.5	116 (79.5-207)	259.8 ± 53.1	5 ± 0.5	998 ± 60.8	15 (3-108.5)
Go-right (11)	42	4.8 ± 0.6	74 ± 8.6	66.8 ± 8.3	181 (88-241)	60.1 ± 5.8	0.6 ± 0.3	n.a	14 (2-80)
Go-silent (21)	21	49.5 ± 4.6	1671 ± 136.4	1628 ± 131	101 (69.5-138.5)	1111 ± 127.7	3.7 ± 0.5	994.3 ± 95.3	15 (3-56)
WSLS (12)	29	146.8 ± 12.3	2290 ± 307.2	2008 ± 293	65 (52.25-90.5)	776.5 ± 223.1	6.3 ± 1.4	3214 ± 368.5	196 (48.75-336.8)

The rule-specific empirical learning criterion is reached if the corresponding strategy sequence length exceeds *learn thresh*, *sequences* is the number of strategy sequences rats experienced while they learned a task rule, y is the trial at which the empirical learning criterion is reached, *perf cp* is the trial at which the performance change point is reached, *seq long* is the longest correct strategy sequence (in trials), x_1 is the trial when the correct strategy was followed for the first time, x_2 is the number of sequences the correct strategy was followed prior to learning, x_3 is a perseveration measure at the strategy level (measured in trials), *om* are the number of omissions. **p<0.01, ****p<10-4, one-way ANOVA or Kruskal-Wallis test (comparison of respective parameter between rules). Source data are provided as a Source Data file.

- *Learning & performance:* analyses were based on the empirical learning criterion and the performance change point (our two measures for learning) from 117/125 rats. N=8 rats (rule: win-stay-lose-shift/1, go-click/7) were excluded because either the empirical learning

criterion was not reached (flat learning curve, N=2) or no significant performance change point (in most cases performance was high from beginning, N=6) could be detected. The longest correct sequence (i.e., the strategy that corresponds to the experimenter-defined rule) served as a measure of performance.

- Win-stay-lose-shift/WSLS: note that learning occurred in 11/12 rats (empirical learning criterion reached) but performance as measured by longest correct sequence was much lower as compared to other rules even though we trained rats for up to 20 days (we only stopped training if ≥50% of trials in a session were explained by detected *win-stay-lose-shift* sequences). Steady-state performance thus even stays lower with extensive training, which may be at least partially explained by probabilistic feedback in this rule. Therefore, the detected performance change points are less meaningful than in other rules.
- The block length of *go-left* sequences corresponding to the empirical learning threshold for the rule go-left was 76 trials (this rule was only used in rats performing multiple switches).
- As a measure for strategy perseveration, we computed how many trials rats need to sort out the most salient strategy (defined as the 10-90%-rise time of the empirical cumulative distribution based on all detected sequences of that strategy). *Go-click* was chosen as the most salient strategy based on the finding that it was tested first across all rules (1 (1-2)). The only exception was the rule go-silent: in this case, we picked the *place* strategy the rat spontaneously used more often (*go-click* is less suitable here because it is the only strategy with consistent negative feedback and thus used less often). Due to the steep learning curve, values for the rules go-click and go-right are not meaningful and thus not reported.

Supplementary Tables 5, 6. Linear regression model based on strategy measures. Related to Fig. 1.

Rats learning a single rule were included in this analysis. The linear regression model was fitted to predict the "learning trial" $y(R)$ for rule R and based on 95/125 rats learning five different rules (alternate, go-dark, go-light, go-silent, win-stay-lose-shift). One rat (rule: win-stay-lose-shift) was excluded because it did not reach learning criterion, 29 rats (rule: one rat learning go-light, all rats learning the rules go-click & go-right) were excluded because the corresponding strategies were so salient for those rats that they almost directly selected them (i.e., in the absence of learning: first trial with correct strategy after 23 (5-61.5) trials). No outliers were removed. Results are provided for robust regression and ordinary least squares regression. Values for R^2 are adjusted for the number of predictors. Source data are provided as a Source Data file.

$$y(R) = \beta_0 + \beta_1 x_1(R) + \beta_2 x_2(R) + \beta_3 x_3(R) + \beta_4 x_4(R) + \epsilon(R), \qquad (1)$$

where,

- x_1 is the trial when the correct strategy was followed for the first time.
- x_2 is the number of sequences the correct strategy was followed prior to learning.
- x_3 is a perseveration measure[1] at the strategy level (Supplementary Table 4).
- x_4 is a categorical variable indicating the target rule.

The MATLAB routine *fitlm* transfers the categorical variable automatically into dummy indicator variables (1, when the rule is the target, experimenter-defined rule; 0, otherwise).

Estimated Coefficients for robust regression:

	Estimate	SE	tStat	pValue
**(Intercept)**	-62.8	95.3	-0.66	0.51
x_1	0.28	0.07	3.95	$1.6*10^{-4}$
x_2	77.52	11.07	7.00	$5.0*10^{-10}$
x_3	0.72	0.06	11.81	$9.0*10^{-20}$
x_{4_2}	120.15	92.39	1.30	0.20
x_{4_4}	415.37	106.8	3.89	$2.0*10^{-4}$
x_{4_7}	-108.2	94.26	-1.15	0.25
x_{4_8}	-1267.8	163.05	-7.78	$1.4*10^{-11}$

Number of observations: 95, Error degrees of freedom: 87, Root Mean Squared Error: 290, R^2: 81.5%, Adjusted R^2: 80.0%, F-statistic vs. constant model: 54.6, p-value = $3.7*10^{-29}$

Estimated Coefficients for ordinary least squares regression:

	Estimate	SE	tStat	pValue
**(Intercept)**	47.95	109.1	0.44	0.66
x_1	0.44	0.08	5.40	$5.8*10^{-7}$
x_2	102.73	12.68	8.10	$3.1*10^{-12}$
x_3	0.41	0.07	5.83	$9.3*10^{-8}$
x_{4_2}	130.6	105.83	1.23	0.22
x_{4_4}	353.79	122.34	2.89	0.0048
x_{4_7}	-45.53	107.97	-0.42	0.67
x_{4_8}	-9.65	186.76	-0.05	0.96

Number of observations: 95, Error degrees of freedom: 87, Root Mean Squared Error: 332, R^2: 77.9%, Adjusted R^2: 76.2%, F-statistic vs. constant model: 43.9, p-value = $6.4*10^{-26}$

Supplementary Table 7. Rat experimental groups used in this study.

Experimental group	Group size [N]	Comment
Rats performing one rule (multidimensional rule-learning task)*	125	alternate N=19, go-click N=17, go-dark N=24, go-light N=21, go-right N=11, go-silent N=21, win-stay-lose-shift N=12
Random reward feedback (multidimensional rule-learning task)*	19	
Multiple rules switches (multidimensional rule-learning task)*	33	dark→ place→ alternate→ random: N=11;<br>dark→ place→ alternate→ go-silent: N=22<br>A subset of these rats was implanted with silicon probes (see below)
Conventional deterministic strategy set-shifting task	16	
Rats with silicon probes implanted into prelimbic prefrontal cortex**	10	N=5 probabilistic set-shift, N=5 multidimensional rule-learning task

*Video analyses were performed for N=86 rats: Rats with randomized reward feedback (N=19 experimentally naïve rats), rats learning the rules alternate or go-silent only (N=19 rats each) and 29 animals performing the multi-rule task (four animals had incomplete video data).

**For one rat performing the multidimensional rule-learning task, only four experimental days are available (the implant was damaged after the fourth session and we decided to sacrifice the animal to be able to determine electrode location). One implanted rat performing the probabilistic set-shift had difficulties to reach the performance threshold for rule switches (18 correct trials out of 20) and we therefore lowered the performance threshold to 16 correct trials out of 20 in later sessions. Since the electrophysiological focus of this study was on neural decoding of behavioral strategies (which are detected independent of task performance), no sessions from that animal were excluded for that reason.

Supplementary Table 8. Pseudorandomized list used for cue presentation in multidimensional rule-learning paradigm.

Visual cue (0/1= left/right visual cue active):

1,0,1,0,1,0,1,0,1,1,1,0,0,1,1,0,0,1,0,0,1,1,0,0,0,1,1,0,1,0,1,0,0,1,1,0,0,1,1,0,0,1,0,1,0,1,1,1,0,0,1,0,
1,1,0,0,1,0,1,1,0,0,1,0,1,0,0,1,1,0,0,1,1,0,0,1,1,0,0,1,1,1,0,0,1,0,1,0,1,0,1,0,0,0,1,0,1,0,1,1,0,1,0,0,
1,1,0,0,1,1,1,0,0,0,1,1,0,0,0,1,1,0,1,1,0,1,0,1,1,0,0,1,0,0,1,1,0,1,1,0,1,0,0,1,0,1,0,0,1,0,1,1,0,1,1,0,
0,1,1,0,1,0,1,0,0,1,0,1,0,0,1,1,0,0,1,1,0,1,0,1,0,0,1,1,1,0,0,1,0,0,1,1,0,1,0,1,1,0,1,0

Auditory cue:

0,1,1,0,0,1,1,1,0,1,0,0,1,0,1,0,1,1,0,1,0,1,1,0,1,0,1,1,1,0,0,0,1,1,0,0,1,1,0,1,0,0,1,1,0,0,1,0,0,1,0,0,
1,0,0,1,1,0,0,1,1,0,0,1,1,0,1,1,0,1,0,0,1,1,0,1,0,0,1,0,1,0,1,0,1,1,1,0,0,1,1,1,0,1,0,0,0,1,1,0,0,1,1,0,
0,1,1,0,1,0,1,0,1,0,0,1,0,1,0,1,0,0,1,0,0,1,1,0,1,0,1,1,1,0,0,1,1,0,1,1,0,0,1,1,0,0,0,1,1,1,0,1,0,0,1,1,
0,0,1,0,0,1,1,1,0,0,0,1,1,0,0,1,0,1,0,1,1,0,0,1,1,0,0,1,0,0,1,0,0,1,1,0,0,1,1,0,1,0,0,1

For the multidimensional rule-learning task in rats and humans, we used a pseudorandomized list for cue presentation (instead of a truly randomized presentation) to avoid reinforcement of non-sensory strategies by chance in short blocks of trials during learning of a sensory rule. We took that measure because rats are prone to use *win-stay-lose-shift* or *place* strategies in operant chambers[13]. The list always started at position one (and re-started there after 200 trials) and has the following properties: a cue is presented max. three times in a row on the same side, all sides (0 vs. 1/left vs. right with active cue) and cue pairs (visual-auditory: 0-1, 1-0, 0-1, 1-1) have the same frequency and no pair is directly repeated.

Supplementary Table 9. Correlation of in-task behavior and CANTAB values. Related to Fig. 5.

	Current paradigm			CANTAB			
	correct trials	omissions	strategies tested	IED EDS errors	IED Pre-EDS errors	SWM Strategy	SSP Span length
**correct trials**	1	-0.21	-0.15	**-0.36***	-0.06	0	0
**omissions**		1	-0.20	**0.50****	0.01	-0.11	-0.17
**strategies tested**			1	0.17	-0.12	**-0.40***	0.03
**IED EDS errors**				1	0.17	0.07	-0.3
**IED Pre-EDS errors**					1	0.17	-0.08
**SWM Strategy**						1	-0.18
**SSP Span length**							1

Correlation matrix (N=31 subjects) showing the link between performance in our task and established markers of executive function as measured using the CANTAB neuropsychological test battery that all subjects completed (Methods). From the current paradigm, we used the number of correct trials (across all rules except for the random rule), the total number of omissions and the number of different strategies tested in the random rule as performance markers. We decided to focus on the following four CANTAB parameters: extra-dimensional shift/EDS errors and Pre-EDS errors from the Intra-Extra Dimensional Set Shift, Spatial Working Memory/SWM strategy, Spatial Span/SSP span length. Since our rule-learning task corresponds to an extra-dimensional shift, we expected to observe a correlation of in-task performance with EDS but not Pre-EDS errors. Moreover, we selected two parameters that focus on different aspects of working memory performance. Working memory tasks require online organization of information and SWM strategy

is a measure for how efficient subjects impose structure on the information held in mind. It has been shown that this executive process can be dissociated from mnemonic components in working memory tasks (as measured by SSP span length)[14]. We hypothesized that in-task performance correlates with the planning but not the short-term memory component of working memory. Significant Pearson correlations shown in the table are in line with these predictions but were not corrected for multiple comparisons (*: $p<0.05$, **: $p<0.01$). Source data are provided as a Source Data file.

Supplementary References

1. Floresco, S. B., Block, A. E. & Tse, M. T. L. Inactivation of the medial prefrontal cortex of the rat impairs strategy set-shifting, but not reversal learning, using a novel, automated procedure. *Behavioural brain research* **190**, 85–96 (2008).
2. Russo, E. *et al.* Coordinated Prefrontal State Transition Leads Extinction of Reward-Seeking Behaviors. *J Neurosci* **41**, 2406–2419 (2021).
3. Toutounji, H. & Durstewitz, D. Detecting Multiple Change Points Using Adaptive Regression Splines With Application to Neural Recordings. *Front Neuroinform* **12**, 67 (2018).
4. Rescorla, R. A. & Wagner, A. R. A theory of Pavlovian conditioning and the\reffectiveness of reinforcement and non-reinforcement. in *Classical Conditioning. 2. Current Research and Theory.* (ed. Black AH, P. W.) 64–69 (Appleton-Century-Crofts, New York, 1972).
5. Rossant, C. *et al.* Spike sorting for large, dense electrode arrays. *Nat Neurosci* **19**, 634–641 (2016).
6. Durstewitz, D., Vittoz, N. M., Floresco, S. B. & Seamans, J. K. Abrupt transitions between prefrontal neural ensemble states accompany behavioral transitions during rule learning. *Neuron* **66**, 438–48 (2010).
7. Meyers, E. M. The neural decoding toolbox. *Front Neuroinform* **7**, 8 (2013).
8. Narayanan, N. S., Kimchi, E. Y. & Laubach, M. Redundancy and synergy of neuronal ensembles in motor cortex. *J Neurosci* **25**, 4207–4216 (2005).
9. Eckstein, M. K. & Collins, A. G. E. Computational evidence for hierarchically structured reinforcement learning in humans. *Proc Natl Acad Sci U S A* **117**, 29381–29389 (2020).
10. Collins, A. G. E., Cavanagh, J. F. & Frank, M. J. Human EEG uncovers latent generalizable rule structure during learning. *J Neurosci* **34**, 4677–85 (2014).
11. Farashahi, S., Rowe, K., Aslami, Z., Lee, D. & Soltani, A. Feature-based learning improves adaptability without compromising precision. *Nat Commun* **8**, 1768 (2017).
12. Koechlin, E. An evolutionary computational theory of prefrontal executive function in decision-making. *Philos Trans R Soc Lond B Biol Sci* **369**, (2014).
13. Evenden, J. L. & Robbins, T. W. Win-Stay Behaviour in the Rat. *The Quarterly Journal of Experimental Psychology Section B* **36**, 1–26 (1984).
14. Owen, A. M., Downes, J. J., Sahakian, B. J., Polkey, C. E. & Robbins, T. W. Planning and spatial working memory following frontal lobe lesions in man. *Neuropsychologia* **28**, 1021–34 (1990).
