# Supplementary material for: Abstract rule learning promotes cognitive flexibility in complex environments across species
Source: Nat Commun. 2025 Jun 25;16:5396. doi: 10.1038/s41467-025-60943-7 (PMC12198366; doi:10.1038/s41467-025-60943-7)
Supplement: Supplementary file 2 — Reporting Summary [file 41467_2025_60943_MOESM2_ESM.pdf]

nature portfolio

Corresponding author(s): Florian Bähner (florian.baehner@zi-mannheim.de)

Last updated by author(s): Jun 4, 2025

Reporting Summary

Nature Portfolio wishes to improve the reproducibility of the work that we publish. This form provides structure for consistency and transparency in reporting. For further information on Nature Portfolio policies, see our Editorial Policies and the Editorial Policy Checklist.

Statistics

For all statistical analyses, confirm that the following items are present in the figure legend, table legend, main text, or Methods section.

n/a	Confirmed	
☐	☒	The exact sample size (*n*) for each experimental group/condition, given as a discrete number and unit of measurement
☐	☒	A statement on whether measurements were taken from distinct samples or whether the same sample was measured repeatedly
☐	☒	The statistical test(s) used AND whether they are one- or two-sided<br>*Only common tests should be described solely by name; describe more complex techniques in the Methods section.*
☐	☒	A description of all covariates tested
☐	☒	A description of any assumptions or corrections, such as tests of normality and adjustment for multiple comparisons
☐	☒	A full description of the statistical parameters including central tendency (e.g. means) or other basic estimates (e.g. regression coefficient) AND variation (e.g. standard deviation) or associated estimates of uncertainty (e.g. confidence intervals)
☐	☒	For null hypothesis testing, the test statistic (e.g. *F*, *t*, *r*) with confidence intervals, effect sizes, degrees of freedom and *P* value noted<br>*Give P values as exact values whenever suitable.*
☒	☐	For Bayesian analysis, information on the choice of priors and Markov chain Monte Carlo settings
☐	☒	For hierarchical and complex designs, identification of the appropriate level for tests and full reporting of outcomes
☐	☒	Estimates of effect sizes (e.g. Cohen's *d*, Pearson's *r*), indicating how they were calculated

Our web collection on statistics for biologists contains articles on many of the points above.

Software and code

Policy information about availability of computer code

Data collection	Operant procedures in rats: custom-made MedStat notation code (MedPC IV, MED Associates, St. Albans, VT, USA)<br>Rat video recordings: Image Acquisition Toolbox, MATLAB, Natick, MA, USA<br>Rat electrophysiological recordings: recordings were performed using freely available software for controlling the RHD USB interface board provided by the manufacturer (Intan Technologies LLC, CA, USA; intantech.com/downloads.html)<br>Human behavioral procedures: custom-made code using Presentation (Version 20.1, Neurobehavioral Systems, Berkeley, CA, USA)<br>Human MEG acquisition: 306-sensor TRIUX MEGIN system (MEGIN, Finland), a signal space separation algorithm implemented in the Maxfilter program provided by the manufacturer was used

Data analysis	Statistical testing: Graphpad Prism (Version 7), IBM SPSS Statistics (Version 29.0.0.0, IBM, Armonk, NY, USA) and MATLAB (R2017a and higher, MATLAB, Natick, MA, USA)<br>Strategy detection algorithm, RL models: custom-written Matlab scripts<br>Change point detection in behavioral time series: MATLAB code is available at https://github.com/htoutounji/PARCS<br>Machine learning-based image analysis: Ilastik-1.2.0, http://ilastik.org/, custom-written Python script (https://www.python.org/), custom-written MATLAB scripts<br>Rat spike sorting: automatic spike sorting with Klusta (https://github.com/kwikteam/klusta), manual curation with Klustaviewa (https://github.com/klusta-team/klustaviewa)<br>Rat neural decoding: Neural Decoding Toolbox (www.readout.info)<br>Human MEG analysis: MATLAB-based toolbox for neuroelectric and neuromagnetic data analysis FieldTrip (https://www.fieldtriptoolbox.org/), MVPA-Light toolbox (https://github.com/treder/MVPA-Light)<br><br>Custom Matlab code (strategy detection algorithm, RL modelling) can be found here: https://doi.org/10.5281/zenodo.15466504

For manuscripts utilizing custom algorithms or software that are central to the research but not yet described in published literature, software must be made available to editors and reviewers. We strongly encourage code deposition in a community repository (e.g. GitHub). See the Nature Portfolio guidelines for submitting code & software for further information.

Data

Policy information about availability of data

All manuscripts must include a data availability statement. This statement should provide the following information, where applicable:

- Accession codes, unique identifiers, or web links for publicly available datasets
- A description of any restrictions on data availability
- For clinical datasets or third party data, please ensure that the statement adheres to our policy

Rat and human data sets used in this study can be accessed via: https://doi.org/10.5281/zenodo.15466504

Research involving human participants, their data, or biological material

Policy information about studies with human participants or human data. See also policy information about sex, gender (identity/presentation), and sexual orientation and race, ethnicity and racism.

Reporting on sex and gender	32 healthy adults without a history of mental disorder (22 females/10 males, median age 24.5, range 19-55 years) were recruited from the local community. Gender was determined based on self-reporting. Since the focus of the study was on establishing a cross-species link with respect to cognitive flexibility mechanisms, no attempt was made to include gender-based analyses. Consent to share individual-level data has been obtained from all but one participant who wanted to restrict the use of data to this study. We therefore cannot provide the information whether individual data points are from male or female subjects.
Reporting on race, ethnicity, or other socially relevant groupings	See above, no attempt was made to include such categories because the focus of the study was to establish a cross-species link with respect to cognitive flexibility mechanisms.
Population characteristics	See above section on sex and gender
Recruitment	Subjects were recruited from the local community using flyers and social media postings of our institute.
Ethics oversight	The study was approved by the local ethics committee (Medizinische Ethik-Kommission II, Heidelberg University, Medical Faculty Mannheim, Germany).

Note that full information on the approval of the study protocol must also be provided in the manuscript.

Field-specific reporting

Please select the one below that is the best fit for your research. If you are not sure, read the appropriate sections before making your selection.

☒ Life sciences ☐ Behavioural & social sciences ☐ Ecological, evolutionary & environmental sciences

For a reference copy of the document with all sections, see nature.com/documents/nr-reporting-summary-flat.pdf

Life sciences study design

All studies must disclose on these points even when the disclosure is negative.

Sample size	216 male Sprague Dawley rats (distributed across experimental groups as outlined in Supplementary Table 7), 32 healthy human subjects. Since this is a new experimental paradigm, effect sizes were unknown and no formal sample-size calculations were possible. We based the choice of sample size based on the rat and human literature on executive functions. Sample sizes are sufficient because the hypothesized differences between experimental groups in rats could be confirmed and evidence for strategy-based learning was also found in the human sample (i.e., statistical power was sufficient).

Data exclusions	Rats: 17 rats were excluded from data analysis either due to incomplete data (hardware/software problems or human error, N=12) or bad signal quality in the recovery period after surgery in the case of implanted rats (N=5). Humans: one subject discontinued participation in the study prematurely; three subjects could not be included in the MEG analysis (two due to strong artifacts, one subject for behavioral reasons).
Replication	Findings on strategy-based learning using behavioral, computational and electrophysiological approaches could be replicated in several cohorts of rats (Supplementary Table 7). Rats learned either one out of eight different rules (behavioral and computational modelling) or multiple consecutive rules (behavioral, computational and electrophysiological correlates of strategy-based learning) in our new rule-learning paradigm. Comparable findings were obtained in a group of rats performing a conventional set-shifting task (behavior, electrophysiology). Moreover, behavioral, computational and electrophysiological correlates of strategy-based learning were also found in a group of healthy human subjects.
Randomization	Rats were randomly allocated to experimental groups. All human subjects were allocated to the same experimental group.
Blinding	Blinding was not necessary in humans (no group comparisons) and not possible in rats. However, experiments using group comparisons in rats were designed to test hypotheses derived from theoretical models and data analysis was thus not exploratory.

Reporting for specific materials, systems and methods

We require information from authors about some types of materials, experimental systems and methods used in many studies. Here, indicate whether each material, system or method listed is relevant to your study. If you are not sure if a list item applies to your research, read the appropriate section before selecting a response.

Materials & experimental systems

n/a	Involved in the study
☒	☐ Antibodies
☒	☐ Eukaryotic cell lines
☒	☐ Palaeontology and archaeology
☐	☒ Animals and other organisms
☒	☐ Clinical data
☒	☐ Dual use research of concern
☒	☐ Plants

Methods

n/a	Involved in the study
☒	☐ ChIP-seq
☒	☐ Flow cytometry
☒	☐ MRI-based neuroimaging

Animals and other research organisms

Policy information about studies involving animals; ARRIVE guidelines recommended for reporting animal research, and Sex and Gender in Research

Laboratory animals	Male Sprague Dawley rats.
Wild animals	This study did not involve wild animals.
Reporting on sex	Research was only conducted in male rats. An in-depth analysis of the general phenomenon of strategy-based learning already required >200 animals and we thus did not attempt a comparison between sexes.
Field-collected samples	The study did not invlove samples collected from the field.
Ethics oversight	All experiments in this study were performed in accordance with national and international ethical guidelines, conducted in compliance with the German Animal Welfare Act and approved by the local authorities (Regierungspräsidium Karlsruhe, Germany).

Note that full information on the approval of the study protocol must also be provided in the manuscript.

Plants

Seed stocks	*Report on the source of all seed stocks or other plant material used. If applicable, state the seed stock centre and catalogue number. If plant specimens were collected from the field, describe the collection location, date and sampling procedures.*
Novel plant genotypes	*Describe the methods by which all novel plant genotypes were produced. This includes those generated by transgenic approaches, gene editing, chemical/radiation-based mutagenesis and hybridization. For transgenic lines, describe the transformation method, the number of independent lines analyzed and the generation upon which experiments were performed. For gene-edited lines, describe the editor used, the endogenous sequence targeted for editing, the targeting guide RNA sequence (if applicable) and how the editor was applied.*
Authentication	*Describe any authentication procedures for each seed stock used or novel genotype generated. Describe any experiments used to assess the effect of a mutation and, where applicable, how potential secondary effects (e.g. second site T-DNA insertions, mosiacism, off-target gene editing) were examined.*
